# Nasal Expiratory Positive Airway Pressure Devices (Provent) for OSA: A Systematic Review and Meta-Analysis

**DOI:** 10.1155/2015/734798

**Published:** 2015-12-21

**Authors:** Muhammad Riaz, Victor Certal, Gaurav Nigam, Jose Abdullatif, Soroush Zaghi, Clete A. Kushida, Macario Camacho

**Affiliations:** ^1^Twin Cities Community Hospital, Templeton, CA 93465, USA; ^2^Department of Otorhinolaryngology, Sleep Medicine Centre, Hospital CUF, 4100-180 Porto, Portugal; ^3^Centre for Research in Health Technologies and Information Systems (CINTESIS), University of Porto, 4200-450 Porto, Portugal; ^4^Clay County Hospital, 911 Stacy Burk Drive, Flora, IL 62839, USA; ^5^Department of Otorhinolaryngology, Hospital Bernardino Rivadavia, Buenos Aires, Argentina; ^6^Department of Head and Neck Surgery, University of California, Los Angeles, CA 90095, USA; ^7^Sleep Medicine Division, Department of Psychiatry and Behavioral Sciences, Stanford Hospital and Clinics, Redwood City, CA 94063, USA; ^8^Division of Otolaryngology, Sleep Surgery, and Sleep Medicine, Tripler Army Medical Center (Tripler AMC), 1 Jarrett White Road, Honolulu, HI 96859, USA

## Abstract

*Objective.* To quantify the effectiveness of nasal expiratory positive airway pressure (nasal EPAP) devices or Provent as treatment for obstructive sleep apnea (OSA).* Methods.* PubMed and six other databases were searched through November 15, 2015, without language limitations.* Results.* Eighteen studies (920 patients) were included. Pre- and post-nasal EPAP means ± standard deviations (M ± SD) for apnea-hypopnea index (AHI) in 345 patients decreased from 27.32 ± 22.24 to 12.78 ± 16.89 events/hr (relative reduction = 53.2%). Random effects modeling mean difference (MD) was −14.78 events/hr [95% CI −19.12, −10.45], *p* value < 0.00001. Oxygen desaturation index (ODI) in 247 patients decreased from 21.2 ± 19.3 to 12.4 ± 14.1 events/hr (relative reduction = 41.5%, *p* value < 0.00001). Lowest oxygen saturation (LSAT) M ± SD improved in 146 patients from 83.2 ± 6.8% to 86.2 ± 11.1%, MD 3 oxygen saturation points [95% CI 0.57, 5.63]. Epworth Sleepiness Scale (ESS) M ± SD improved (359 patients) from 9.9 ± 5.3 to 7.4 ± 5.0, MD −2.5 [95% CI −3.2, −1.8], *p* value < 0.0001.* Conclusion.* Nasal EPAP (Provent) reduced AHI by 53.2%, ODI by 41.5% and improved LSAT by 3 oxygen saturation points. Generally, there were no clear characteristics (demographic factors, medical history, and/or physical exam finding) that predicted favorable response to these devices. However, limited evidence suggests that high nasal resistance could be associated with treatment failure. Additional studies are needed to identify demographic and polysomnographic characteristics that would predict therapeutic success with nasal EPAP (Provent).

## 1. Introduction

Obstructive sleep apnea (OSA) is a chronic disorder affecting millions of Americans with an escalating prevalence between 3% and 10% in middle-aged adults [1]. Positive airway pressure (PAP) is considered as the most effective treatment for OSA [2]. Consistent PAP device usage leads to improvement or even complete alleviation of daytime symptoms and PAP therapy also mitigates cardio- and neurovascular complications of untreated sleep apnea [3]. Nevertheless, poor PAP adherence is considered as a major barrier for optimal OSA treatment [4]. Therefore alternative treatment options have been investigated over the years to include upper airway surgeries, hypoglossal nerve stimulators, oral appliances, and Winx oral devices as well as more recently Provent therapy which are also known as nasal expiratory positive airway pressure (nasal EPAP) devices.

Nasal EPAP device consists of disposable one-way resister valves, which are placed over the nostrils with an adhesive tape. These valves operate by utilizing the patient's own breathing to create a positive end-expiratory pressure with minimal inspiratory resistance. This high-end-expiratory pressure leads to upper airway dilation with subsequent tracheal traction and increased lung volumes during exhalation, thereby making the upper airway more resistant to ensuing inspiration [5–7]. Several studies have evaluated the efficacy of nasal EPAP given high noncompliance rate of PAP therapy due to either intolerance or difficulty associated with its use during travelling, in wilderness, and in places with lack of electricity [8]. The objective of this systematic review and meta-analysis is to quantitatively evaluate the effectiveness of nasal EPAP on polysomnography variables and sleepiness in OSA patients.

## 2. Methods

This study is exempt from Institutional Review Board (IRB) protocol being a systematic review and meta-analysis. Electronic databases were searched initially from inception through February 1, 2015, with an update through November 15, 2015, and included PubMed, Scopus, Embase, Google Scholar, Web of Science, CINAHL, and The Cochrane Library. The study designs that were included in this review are posters, abstracts, conference proceedings, case reports, case series, cohort, and randomized trials. The searches were conducted by combining selected MeSH terms, keywords, and phrases to achieve maximum sensitivity. An example of search strategy used in PubMed is as follows: (((“sleep” OR (“Sleep Apnea, Obstructive”[Mesh])) OR ((“Therapeutics”[Mesh]) OR (“Complementary Therapies”[Mesh]))) AND ((“Provent”) OR (“nasal expiratory positive airway pressure” OR (“nasal EPAP”)))). Conference proceedings, nasal EPAP patent publications, and references of relevant articles were also searched to minimize the risk of missing potentially relevant publications. Also clinical trial websites were searched using appropriate terms; only one clinical trial was found which is recently completed but not published yet.

Data was abstracted in blinded manner, and reviewers (MR and MC) agreed on the included studies. Articles were selected using preset inclusion and exclusion criteria and studies were included for the review by the consensus of authors (MR and MC). Inclusion criteria, (1) adult OSA patients who underwent treatment with nasal EPAP or Provent therapy with quantitative outcome data such as apnea-hypopnea index (AHI), quality of life questionnaires, or Epworth Sleepiness Scale (ESS) before and after treatment, (2) studies with the objective of evaluating the effectiveness of nasal EPAP, and (3) all languages, were included. Exclusion criteria were as follows: (1) studies using Theravent as a snore prevention device, (2) patients under 18 years of age, or (3) no clear outcomes such as AHI and ESS reported. The meta-analysis was only performed for those studies, which reported means and standard deviations. The corresponding authors for studies reporting median values were contacted twice, and if the means and standard deviations could not be obtained, then the studies were included in the systematic review but were excluded from this meta-analysis.

The primary outcomes reviewed in the meta-analysis included the AHI, respiratory disturbance index (RDI), oxygen desaturation index (ODI), ESS, and quality of life questionnaires to assess the efficacy of nasal EPAP. Secondary outcomes included adherence or any other outcome reported by included studies. The National Institute for Health and Clinical Excellence (NICE) was used to assess the quality of studies. The Preferred Reporting Items for Systematic Reviews and Meta-Analyses (PRISMA) statement was utilized throughout this systematic review and meta-analysis [9].

### 2.1. Statistical Analysis

Statistical evaluation was performed using Review Manager (REVMAN) software version 5.3 (Copenhagen: The Nordic Cochrane Centre, The Cochrane Collaboration, 2014). The pre- and post-nasal EPAP means, standard deviations (SD), mean differences (MD), 95% confidence intervals (CI), and *p* values were calculated using the IBM Statistical Package for Social Sciences (SPSS) software, version 20.0 (Armonk, New York, USA). Combined mean differences and 95% CI were calculated for studies reporting means and SD. Combined mean differences were not calculated for studies who did not provide standard deviations or median values; instead data was manually entered into the table. The null hypothesis for this study was that there is no difference between polysomnographic outcomes and ESS pre- and post-nasal EPAP therapy. Random effects modeling was used for analysis. Bias was analyzed by evaluating the funnel plots created by REVMAN. Inconsistency was evaluated using *I* ^2^ statistic (low: 25%, moderate: 50%, and high: 75%) [10]. The Cochran *Q* statistic *p* value ≤ 0.10 was considered statistically significant heterogeneity based on published guidelines [11]. For each variable evaluated (AHI, ESS, etc.) a sensitivity analysis was performed if there was inconsistency or heterogeneity in order to determine which study was the cause; this was performed by using REVMAN and removing one study at a time until no inconsistency or heterogeneity was present.

## 3. Results

**Figure 1:**
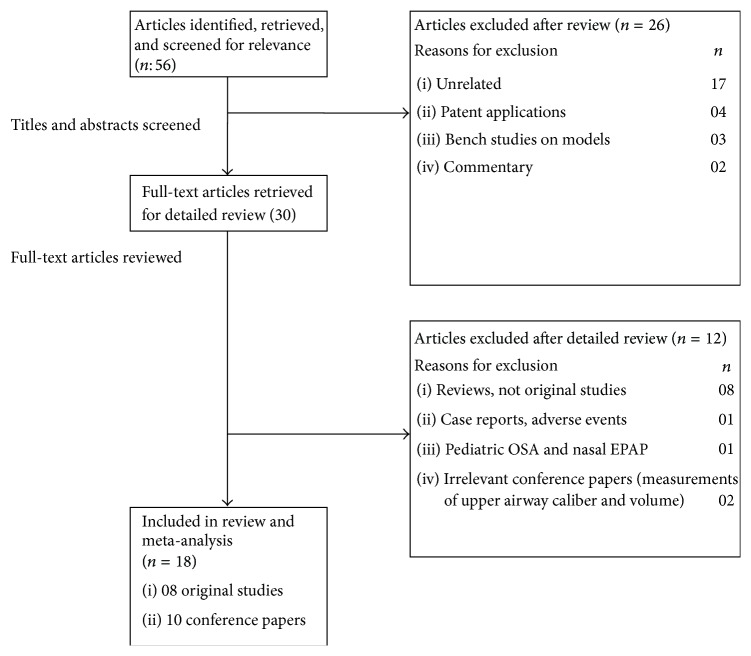
Nasal EPAP devices study selection flowchart.

The searches yielded a total of fifty-six articles after exclusion of duplicates, which were screened. Of these articles, thirty of them were potentially relevant and the full-text versions were downloaded for detailed evaluation (Figure 1). Eight of these were review articles discussing nasal EPAP alternative as treatment for OSA, and one was a case report discussing the emergence of complex sleep apnea with nasal EPAP use. After detailed review, the authors agreed that eighteen studies (eight original studies [12–19] and ten conference papers [20–29]) met the inclusion and exclusion criteria. The combined studies had 920 patients, mean age 50.2 ± 12.7 years, with an average body mass index (BMI) of 32.2 ± 6.7 kg/m^2^. The earliest published study was by Colrain et al. in 2008 and Friedman et al. published the most recent study in 2015. The outcomes analyzed by these studies included AHI, ODI, minimum oxygen saturation (SPO_2_), mean SPO_2_, percent of total sleep time (%TST) with SPO_2_ < 90%, critical closing pressure (*P*_crit_), end tidal CO_2_ (EtCO_2_), lung volumes, blood pressure (BP), and quality of life questionnaires including Pittsburgh Sleep Quality Index (PSQI), Functional Outcomes of Sleep Questionnaire (FOSQ), and ESS.

All original studies underwent a quality assessment using the NICE quality assessment tool, and most of these studies were prospective with one dual-center and one multicenter randomized clinical trial (Table 1). The study quality assessment is presented in Table 2. Overall, the quality of original included studies was high and majority of them met 6-7 out of 8 criteria items evaluated by the quality assessment tool.

## 4. General Characteristics of Included Studies

The follow-up data was highly variable ranging from overnight study to a 12-month follow-up. Six original studies reported nasal EPAP to be effective in reducing AHI and other OSA related parameters. One randomized dual-center placebo-controlled trial failed to show efficacy of nasal EPAP and the majority of patients in this trial had elevated AHI and ESS (Table 3) and LSAT and ODI (Table 4). This study by Rossi et al. included OSA patients who were previously optimally treated on CPAP and were randomized into CPAP, Placebo-Provent, and Provent for 2 weeks and then were tested for OSA measures including AHI, ODI, and ESS as well as diastolic blood pressure. These patients were optimally treated with CPAP prior to randomization. This study reported higher residual AHI as well as diastolic blood pressure in Placebo and Provent group after CPAP withdrawal. Studies reporting adherence had an overall high nasal EPAP use, ranging between 80 and 94%.

## 5. Treatment Effect Data

### 5.1. Apnea-Hypopnea Index

**Figure 2:**
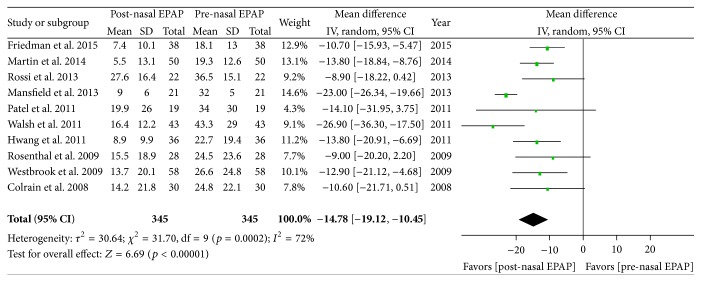
Pre- and post-nasal EPAP therapy outcomes for apnea-hypopnea index (events per hour), mean difference. SD: standard deviation; CI: confidence interval; nasal EPAP device: nasal expiratory positive airway pressure device (Provent).

**Figure 3:**
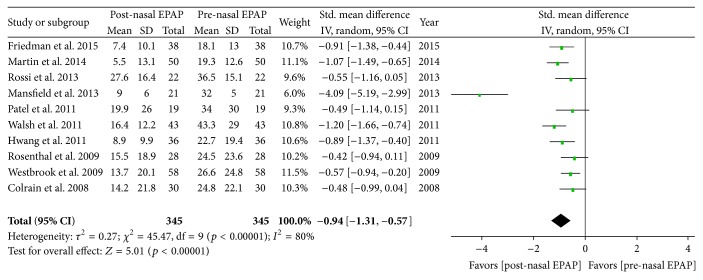
Pre- and post-nasal EPAP therapy outcomes for apnea-hypopnea index (events per hour), standardized mean difference. SD: standard deviation; CI: confidence interval; nasal EPAP device: nasal expiratory positive airway pressure device (Provent).

Polysomnography outcomes for nasal EPAP in 345 patients demonstrated that the AHI decreased from an overall mean (M) ± standard deviation (SD) in 345 patients from 27.32 ± 22.24 to 12.78 ± 16.89 events/hr (relative reduction = 53.2%); see Table 3. A subanalysis for these studies was performed and the AHI mean difference was −14.78 events/hr [95% CI −19.12, −10.45], overall effect *Z* = 6.69, *p* value < 0.00001, *Q* statistic *p* value = 0.0002 (significant heterogeneity), and *I* ^2^ = 72% (high inconsistency); see Figure 2. The funnel plot for AHI MD was scattered and only moderately distributed into an inverted funnel shape suggesting moderate publication bias. The sensitivity analysis demonstrated that the studies by Mansfield et al. and Walsh et al. contributed to the heterogeneity and inconsistency and, after their removal from the meta-analysis, there was no significant heterogeneity (*Q* statistic *p* value = 0.97) and no inconsistency (*I* ^2^ = 0%). For all 345 patients, the AHI standardized mean difference (SMD) was −0.94 [95% CI −1.31, −0.57] (large magnitude of effect), overall effect *Z* = 5.01, *p* value < 0.00001, *Q* statistic *p* value < 0.00001 (significant heterogeneity), and *I* ^2^ = 80% (high inconsistency); see Figure 3.

### 5.2. Oxygen Desaturation Index

**Figure 4:**
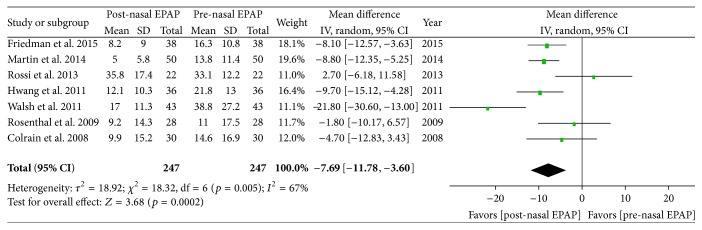
Pre- and post-nasal EPAP therapy outcomes for oxygen desaturation index (events per hour), mean difference. SD: standard deviation; CI: confidence interval; nasal EPAP device: nasal expiratory positive airway pressure device (Provent).

Polysomnography outcomes for nasal EPAP demonstrated that the ODI decreased from an overall M ± SD of 21.2 ± 19.3 to 12.4 ± 14.1 events/hr (relative reduction = 41.5%); see Table 4. A subanalysis using random effects modeling was performed for seven studies (247 patients) in which M ± SD were reported, and the ODI mean difference was −7.69 events/hr [95% CI −11.78, −3.60], overall effect *Z* = 3.68, *p* value = 0.0002, *Q* statistic *p* value = 0.005 (significant heterogeneity), and *I* ^2^ = 67% (moderate inconsistency); see Figure 4. The funnel plot for ODI MD was distributed evenly into an inverted funnel shape, suggesting no publication bias. The sensitivity analysis was performed and studies by Walsh et al. and Rossi et al. were found to be the sources of heterogeneity; after the removal of those two studies, there was no significant heterogeneity (*Q* statistic *p* value = 0.51) and no inconsistency (*I* ^2^ = 0%). For all 247 patients, the ODI SMD was −0.58 [95% CI −0.91, −0.25] (medium magnitude of effect), overall effect *Z* = 3.42, *p* = 0.0006, *Q* statistic *p* value = 0.004 (significant heterogeneity), and *I* ^2^ = 69% (moderate inconsistency).

### 5.3. Lowest Oxygen Saturation

**Figure 5:**
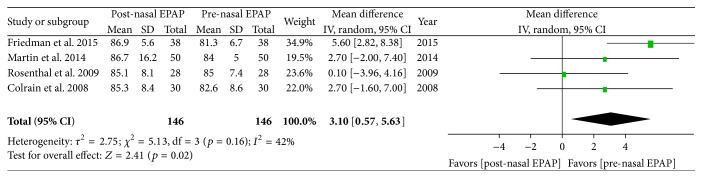
Pre- and post-nasal EPAP therapy outcomes for lowest oxygen saturation (percent), mean difference. SD: standard deviation; CI: confidence interval; nasal EPAP device: nasal expiratory positive airway pressure device (Provent).

Polysomnography outcomes for nasal EPAP demonstrated that the minimum SPO_2_ improved from an overall M ± SD of 83.2 ± 6.8% to 86.2 ± 11.1% (3-point oxygenation increase); see Table 4. A subanalysis using random effects modeling was performed for four studies (146 patients) in which M ± SD were reported, and the minimum SPO_2_ mean difference was 3.10 oxygen saturation points [95% CI 0.57, 5.63], overall effect *Z* = 2.41, *p* value = 0.02, *Q* statistic *p* value = 0.16 (no statistically significant heterogeneity), and *I* ^2^ = 42 (low inconsistency) (Figure 5). Exclusion of the study by Friedman et al. resulted in no significant heterogeneity (*Q* statistic *p* value = 0.61) and no inconsistency (*I* ^2^ = 0%). The funnel plot was not performed given that there were only four studies. The minimum SPO_2_ SMD was 0.37 [95% CI 0.00, 0.73] (small magnitude of effect), overall effect *Z* = 1.97, *p* value = 0.05, *Q* statistic *p* value = 0.07 (significant heterogeneity), and *I* ^2^ = 58% (moderate inconsistency).

### 5.4. Epworth Sleepiness Scale

The sleepiness questionnaire for pre- and post-nasal EPAP demonstrated that the ESS improved from an overall M ± SD of 9.94 ± 5.29 to 7.42 ± 4.98, *p* value < 0.0001; see Table 3. A subanalysis using random effects modeling was performed for five studies (359 patients) in which M ± SD were reported, and the ESS mean difference was −2.61 [95% CI −3.29, −1.94], overall effect *Z* = 7.55, *p* value < 0.00001, *Q* statistic *p* value = 0.64 (no significant heterogeneity), and *I* ^2^ = 0% (no inconsistency). The funnel plot for ESS MD was fairly evenly distributed into an inverted funnel shape, suggesting no publication bias. For all 359 patients, the ESS SMD was −0.52 [95% CI −0.71, −0.33] (medium magnitude of effect), overall effect *Z* = 5.43, *p* value < 0.00001, *Q* statistic *p* value = 0.30 (no significant heterogeneity), and *I* ^2^ = 18% (no inconsistency).

### 5.5. Snoring

Snoring was not assessed by all studies but reduction in snoring was uniformly seen in studies that assessed snoring (i.e., Colrain et al., Friedman et al., and Kryger et al.).

## 6. Discussion

Not all OSA treatments modalities are effective in controlling sleep apnea or improving quality of life as considerable variation exists among therapeutic modalities with respect to reductions in obstructive respiratory events [30, 31]. This systematic review and meta-analysis investigated the effectiveness of nasal EPAP and found it to be a promising addition to the existing therapeutic treatment modalities for OSA. There are six main findings of this systematic review and meta-analysis worth noting.

First, the majority of studies (including most conference abstracts) demonstrated an improvement in polysomnography respiratory parameters such as AHI, ODI, and LSAT. Overall, nasal EPAP (Provent) reduced AHI by 53.2%, ODI by 41.5% and improved LSAT by 3 oxygen saturation points. Although AHI was reduced for most studies, the AHI reductions are generally less as compared to CPAP. It is well established that CPAP generally reduces AHI to less than 5 events/hr [4]. The results of nasal EPAP are however somewhat comparable for oral appliances or less invasive surgical procedures which are less effective than CPAP [32, 33]; nonetheless no head to head comparison has been done. Previous studies have compared oral appliances and surgeries with CPAP but studies for nasal EPAP have only compared it to CPAP. Perhaps future studies comparing nasal EPAP with both invasive/less invasive surgeries and oral appliances could be helpful to establish the success rate in each group given that optimal treatment of OSA is the key to prevent cardiovascular complications as even low AHI or mild OSA has been linked with cardiovascular outcomes [34].

Second, there were differences in study design and the length of follow-up which make it difficult to generalize the potential true long-term effectiveness of nasal EPAP. There was only one study (Kryger et al.) with 12-month follow-up which demonstrated maintenance of therapeutic effects in terms of AHI reduction, snoring, and subjective daytime sleepiness. Nevertheless, majority of studies have variable and inconsistent response to treatment with nasal EPAP and longer duration was inversely proportional to residual AHI for unclear reasons. This inconsistent response could be due to the fact that nasal EPAP increases lung volumes by creating positive airway pressure rather than exerting direct pressure at the upper airway like PAP devices. As OSA can be due to the collapse at any airway level, nasal EPAP may not be able to generate enough airway pressure to overcome airway collapse at all levels. In the aforementioned studies, different nasal EPAP devices with varying degrees of expiratory resistance were used without any significant differences in outcomes. The elimination of SDB is dependent on sustained end-expiratory pressures generated by these valves during different stages of sleep; therefore, it is likely that this end-expiratory pressure will be different from individual to individual with respect to different positions, stages of sleep, mouth breathing, nasal obstruction, and so forth. This was objectively demonstrated by Patel et al., where end-expiratory positive pressure varied widely among different nasal EPAP responders (5–23 cm of water). Therefore, it is possible to titrate such patients in sleep lab to determine the lowest effective pressure in different stages/positions of sleep, which can help select an appropriate resistance for nasal EPAP device. Further studies are needed to explore this hypothesis.

Third, there was no clear predictor (demographic factors, medical history, or physical exam findings) as to which patients will respond most favorably to this device. Patel et al. specifically examined demographic factors that could predict therapeutic response to nasal EPAP. In their study, no significant association was found between the degrees of OSA severity and general characteristics of patients including age, BMI, gender, sleep stage dependent SDB, therapeutic CPAP levels, lung volumes, and *P*_crit_ values. Friedman et al. demonstrated that nasal EPAP may be useful in patients without significant nasal obstruction and daily nasal symptoms. Nevertheless, it is quite possible that patients with positional sleep apnea or those with only mild to moderate OSA or patients without significant nasal obstructions at baseline might benefit the most from this therapy. Treatment was not as successful in patients with severe OSA (Colrain et al.) or in those who had low baseline LSAT along with higher AHI (Patel et al., Berry et al., and Rosenthal et al.). An important confounder is that most studies had stringent inclusion and exclusion criteria and patients with severe comorbidities were also excluded which makes it more difficult to determine which patient profile best suites this therapy. Therefore, additional studies are needed not only to confirm which patient characteristics lead to a more favorable response, but also to stratify treatment effect on OSA related chronic comorbid diseases.

Fourth, Rossi et al. demonstrated a higher residual AHI as well as diastolic blood pressure in Placebo and Provent group after CPAP withdrawal. The high residual AHI in both arms of this study (Provent and Placebo-Provent) could be related to recruitment of subjects with higher baseline OSA severity (mean AHI of 38 events/h), when compared to Berry et al.'s study (median AHI 13.8 events/hr in nasal EPAP versus 11.1 events/hr in sham) that showed preferable improvement with Provent therapy alone. Although the two arms in Rossi et al. study were very similar, the residual AHI and ODI after CPAP treatment as well as the four-night withdrawal rebound ODI values were slightly favorable in the Placebo-Provent arm compared to Provent arm. Also, it was not stated if patients had statistically significant difference in proportion to patients with positional OSA between the Placebo-Provent and Provent arm since this can independently influence residual AHI based on sleep position adopted during the night of sleep study.

Fifth, generally adherence was high for most studies that collected data for adherence (e.g., 84.2% in the study by Friedman, 80% by Wash, 88.2% by Berry, 89.3% by Kryger, 94% by Rosenthal, and 99% by Rossi et al.). Most studies did not report any serious adverse effects; however minor adverse events were common as much as in 42% of patients based on the longest duration study by Kryger et al. On searching literature, only one case report of treatment emergent central sleep apnea with nasal EPAP device was identified [35]. Common side effects observed in most studies include difficulty breathing, exhaling, and sleeping, dry mouth, nasal congestion/drainage/discomfort/itching, insomnia, and headache. Despite these adverse effects, adherence rates were not altered. It is however possible that this adherence might be artificially high as data was provided by subjective reports as compared to PAP where data downloads reflect objective adherence. Additionally, there was only one pediatric study which evaluated the efficacy of nasal EPAP in children 8–16 years old [36]. This was a small (14 subjects) clinical trial which reported significant improvement in AHI (nasal EPAP 0.6 versus placebo 4.2) but 3 subjects did not improve and 2 had worsening of OSA. Given that nasal EPAP has very limited data for children, it should be used cautiously, as other treatment options such as adenotonsillectomy, palate expansion, allergy management, and PAP therapy remain the gold standard.

Sixth, additional research is needed. Because it is not clear why some patients responded better to nasal EPAP than others, the stratification of patients based on additional findings or testing would be helpful. Recently, drug induced sleep endoscopy (DISE) studies have demonstrated that the anatomic location and the physiological pattern of collapse influence the baseline severity of OSA and the response to a particular treatment modality. About two-thirds of patients with OSA have a multilevel collapse, with the most common combination being palatal and tongue based collapse [37]. Nasal EPAP generates end-expiratory pressure leading to increase in functional residual capacity and tracheal traction and a trend towards increase in upper airway cross-sectional area [5]. On the other hand, expansion of the velopharyngeal airway volume during sleep is associated with improvement in OSA with use of oral appliances or oral pressure therapy [38, 39]. Could the variable efficacy of nasal EPAP be related to its limited ability to overcome palatal and tongue base collapse, while having a favorable response in individuals with predominance of hypopharyngeal collapse? In the future, DISE and upper airway MRIs during sleep in subjects with OSA while using nasal EPAP may provide substantial information to explain such heterogeneity in treatment response. Another important variable that needs additional research is the effect of nasal cavity (i.e., inferior turbinate size [40], nasal septal deviations, and nasal septal perforations) and nasopharyngeal exam (adenoid hypertrophy) findings.

## 7. Limitations

The authors in this systematic review tried their best to identify all published as well as grey literature related to nasal EPAP; however it is possible that we failed to identify all relevant studies. It is also quite likely that studies that did demonstrate beneficial effect of nasal EPAP were never made for publication. Not all studies reported the same variables or means and hence were not included in the meta-analysis. The largest study by Berry et al. was not used for pooling random effects given that it did not report mean or standard deviations and instead the median data was provided (the corresponding author responded to our emails but raw data was not available). Similarly, many other studies reported data in median values, hence excluded from the meta-analysis portion of this review (Tables 3 and 4). Long-term follow-up data is limited for most studies with only two studies having 3-month and 12-month data. Data regarding pre- and posttreatment with nasal EPAP and BMI changes were not available for most studies. It is important to note that higher BMI is associated with complete concentric palatal collapse [37] and nasal EPAP may not be able to overcome the palatal collapse in all patients with same OSA severity but with different BMI. Given that pre- and post-BMI data is not available for most studies, it is possible that weight loss during the study period could have influenced the final outcome at the end of study. Many of the studies were industry funded; hence results are to be interpreted with caution.

## 8. Conclusion

Although nasal EPAP does not completely eliminate OSA, there is an improvement in OSA outcomes based on polysomnography and questionnaires (quality of life and sleepiness). The benefits include that the devices are highly portable and easy to use and there are no reported major side effects or complications reported from the use. For that reason, nasal EPAP might be an alternative for certain OSA patients who are either intolerant to PAP or in unusual circumstances without electricity or short trips away from home, and for those with mild or position dependent OSA without concurrent chronic medical problems. Further studies are needed to evaluate long-term efficacy and delineate clinical and polysomnographic profiles of patients who would be best suited for this therapy.

## Figures and Tables

**Table 1:** General characteristics of original studies.

Author, year, country	Study type, methods	Number of patients	Duration of treatment	Demographics (M/F)	Average/mean age (years), M/F	Average/mean BMI M/F	OSA severity	Main findings/treatment duration effect
Colrain et al., 2008 [12] USA	(i) Prospective (ii) 30 subjects randomly divided into two groups (16 & 14) treated versus nontreated with n-EPAP (iii) No placebo nasal device (iv) Resistance of device: 60–90 cm H20 sec/Liter	30 24 OSA and 6 primary snorers	1 night	20/10	50.1 ± 9.8/49.0 ± 12.9	28.2 ± 4.0/27.0 ± 5.34	Mild to severe AHI (5.1–83.8)	AHI and ODI decreased, minimum SPO_2_ increased significantly

Patel et al., 2011 [13] USA	(i) Prospective study (ii) Two-night PSG, one with n-EPAP and another with CPAP to check for efficacy and identify predictors of response and possible mechanisms for n-EPAP (iii) Resistance of device: 80 cm H20 sec/Liter	20 (19 included in final analysis)	2 nights	15/5	54.3 ± 12.0	33.5 ± 5.6	Mild to severe AHI with 4% desaturation (34 ± 30/h)	(i) RDI was reduced significantly (ii) Demographics, lung volumes, *P*_crit_, PSG measures are not good predictors of therapeutic success (iii) Positional OSA is a better predictor

Rossi et al., 2013 [14] UK, Switzerland	(i) Prospective (ii) Randomized, dual-center, parallel, placebo-controlled clinical trial (iii) Comparison between Placebo-Provent, Provent, and CPAP after 2-week therapy of each treatment modality	67	~2 weeks	53/14	*CPAP*: 64.4 ± 7.7 *Provent*: 63.2 ± 8.7 *Placebo*: 59.7 ± 12.5	*CPAP*: 35.1 ± 6.0 *Provent*: 33.4 ± 6.6 *Placebo*: 33.0 ± 6.2	Moderate to severe	*@2 weeks*: ODI went up to 35.8 ± 17.4, AHI to 27.6 ± 16.4 from 4.3 ± 5.1 & 2.4. ± 2.4 in Provent, respectively *@2 weeks*: ODI went up to 28.2 ± 18.3, AHI to 24.2 ± 16.8 from 4.3 ± 5.1 & 2.4. ± 2.4 in Placebo-Provent, respectively

Friedman et al., 2015 [15] USA	(i) Prospective (ii) Two-arm pilot study *Arm 1*: no nasal symptoms and <50% nasal obstruction *Arm 2*: occasional nasal symptoms and 50%–80% nasal obstruction	38	1 mo.	54–76% males	*Arm 1*: 51.5 ± 11.8 *Arm 2*: 51.3 ± 11.6	*Arm 1*: 29.4 ± 5.0 *Arm 2*: 30.3 ± 3.7	Mild to moderate	*Arm 1*: 43.3% failure rate, overall 45.2% cured and 9.7% treatment success *Arm 2*: failed treatment

Rosenthal et al., 2009 [16] USA	(i) Prospective, multicenter trial (ii) 4-night protocol, 1-night baseline PSG, 3 nights with device of varying expiratory resistance (50, 80, and 110 cm H20 sec/Liter) (iii) Final PSG after 30-day device use	28	1 mo.	22/6	49.8 ± 10.2	30.1 ± 5.9	Mild to moderate	*@3 initial treatment nights*: 59% response rate in reducing AHI to 50% (13.5 ± 18.7 versus control 24.5 ± 23.6) (*p* < 0.001) *@1 month*: 41% (15.5 ± 18.9 versus control as above) (*p* = 0.001)

Walsh et al., 2011 [17] USA	(i) Prospective study (ii) Three-night protocol with PSG1 without n-EPAP, PSG2 with n-EPAP at 10 days following PSG1, and PSG3 with n-EPAP after 5-6 weeks of PSG2 (iii) Resistance of device: 50, 80 cm H20 sec/Liter	43	~2 mo.	27/16	53.7 ± 10.9	34.9 ± 6.7	Moderate to severe (43.3 ± 29.0/h)	(i) AHI at PSG1 31.9 ± 19.8, PSG2 11.0 ± 7.9 & PSG3 16.4. ± 12.2 (ii) AHI at PSG2 & PSG3 was decreased; however AHI slightly increased at PSG3 compared to PSG2

Berry et al., 2011 [18] USA	(i) Prospective, randomized, double-blinded, multicenter, parallel group, sham-controlled trial (ii) Comparison of n-EPAP on & off versus sham device over 3 months (iii) Resistance of device: 80 cm H20 sec/Liter	229	3 mo.	*Device*: 65.5% males *Sham*: 71.4% males	*Device*: 47.7 ± 13.4 ITT *Sham*: 46.8 ± 12.0 ITT *Device*: 49 ± 13.1 mITT *Sham*: 47.3 ± 12.3 mITT (Age difference nonsignificant in both groups)	*Device*: 32.6 ± 7.0 *Sham*: 33.8 ± 6.5 *Device*: 32.8 ± 6.7 mITT *Sham*: 34.6 ± 6.6 mITT (BMI difference nonsignificant in both groups)	Mild to severe (mainly mild to moderate) Device: 5.3 ± 22.6 Sham: 4.8 ± 21.8	*@1 week*: median AHI change 52.7% (ITT) & 55.1% (mITT) (*p* < 0.001) *@3 months*: median AHI change 42.7% (ITT) & 42.8% (mITT) (*p* < 0.001) *@1 week*: median ODI change 43.2% (mITT) (*p* < 0.001) *@3 months*: median ODI change 35.2% (mITT) (*p* < 0.025) *@3 months*: ESS change 9.9 ± 4.7 to 7.2 ± 4.2 (*p* < 0.0001) in NEPAP versus sham 9.6 ± 4.9 to 8.3 ± 5.1 (*p* = 0.001). In mITT ESS decreased in both as well

Kryger et al., 2011 [19] USA	(i) Prospective, multicenter, single arm, open labeled extension trial of 3-month n-EPAP versus sham study (ii) Comparison of n-EPAP on & off versus sham device over 12 months (iii) Resistance of device: 80 cm H20 sec/Liter	34	12 mo.	63.4% M	50.1 ± 13.6	32.5 ± 7.5	Mild to severe	*@12 mo.*: median AHI reduced to 4.7 versus baseline 15.7 (71.3% success rate) (i) ODI decreased to 7.6 versus baseline 12.6 (ii) ESS decreased to 6 versus baseline 11

~ No strict follow-up was aimed or short-term follow-up or no follow-up. Most studies in this category completed assessment within the above-mentioned time periods.

n-EPAP: nasal EPAP (Provent); CPAP: continuous positive airway pressure; PSG: polysomnogram; ESS: Epworth Sleepiness Scale; ITT: intention to treat; mITT: modified intention to treat.

**Table 2:** General characteristics of included studies.

Study quality assessment^*∗*^											
Author, year, country	Inclusion criteria	Exclusion criteria	Outcomes analyzed	1	2	3	4	5	6	7	8
Colrain et al., 2008 [12] USA	(i) 18 years & up (ii) People who snore or gasp or with apnea reported by their bed partners: selected from community following a telephone screen (iii) Clinic patients following PSG	BMI >35 Kg/m^2^, PAP users, URI symptoms, severe nasal allergies, sinusitis, nasal airflow restriction reported by patients, h/o nose surgery or trauma, insomnia or narcolepsy, or PLMD patients, serious medical problems including respiratory failure, heart failure, stroke, cancer, and MI. Females who are pregnant or planning to be pregnant	AHI, ODI, min SPO_2_, %TST SPO_2_ > 90, %TST snoring, questions related to comfort, ease of breathing via nose, comparison to PAP if patients previously used PAP, and sleep architectures including sleep stages on PSG	Yes	Yes	Yes	Yes	Yes	No	Yes	Yes

Rosenthal et al., 2009 [16] USA	Adults with snoring or witnessed apnea or those who are diagnosed with OSA	Prior PAP use, severe illness including cancer, CHF, COPD, and other coexistent sleep disorders, and sinusitis or severe nasal allergies or nasal blockage	AHI, ODI, PSQI, ESS, and sleep architectures including sleep stages on PSG	Yes	Yes	Yes	Yes	Yes	No	Yes	Yes

Berry et al., 2011 [18] USA	Newly diagnosed or untreated OSA patient with prestudy AHI ≥ 10 and age ≥ 18 years	H/o upper airway surgery, nasal obstruction or chronic nasal decongestant use, severe nocturnal oxygen desaturations, prior PAP or oral device use, sedative or neuromedications which affect alertness, serious or uncontrolled chronic illnesses, and other comorbid sleep disorders	AHI, ODI, SPO_2_ < 90%, ESS, and sleep architectures including sleep stages, effect of position on sleep stages, REM and non-REM AHI, and arousal index on PSG	Yes	Yes	Yes	Yes	Yes	Yes	Yes	Yes

Kryger et al., 2011 [19] USA	Patients with ≥ 50% reduction in AHI or AHI of < 10 on 3-month n-EPAP on PSG or 1-week device off PSG n-EPAP use of ≥ 4 h/night and ≥ 5 nights/week on average 3-month trial of n-EPAP versus sham study	Same criteria as for original n-EPAP versus sham study. H/o upper airway surgery, nasal obstruction or chronic nasal decongestant use, severe nocturnal oxygen desaturations, prior PAP or oral device use, sedative or neuromedications which affect alertness, serious or uncontrolled chronic illnesses, and other comorbid sleep disorders	AHI, ODI, SPO_2_ < 90%, ESS, snoring, and sleep architectures including sleep stages, effect of position on sleep stages, REM and non-REM AHI, and arousal index on PSG	Yes	Yes	Yes	Yes	Yes	No	Yes	Yes

Patel et al., 2011 [13] USA	OSA patients with AHI with 4% desaturation > 5/hr	Patients with significant nasal obstruction, central apnea, CHF, neuromuscular disease, hypoventilation, elevated serum bicarbonate or arterial PCO_2_, and unexplained desaturations on baseline PSG	*P*_crit_, EtCO_2_ in supine and lateral positions in sleep and wakefulness during PSG, awake lung volumes in sitting, supine, and lateral positions including FEV1, FVC, FRC, ERV, TLC, and FEV1/FVC, and sleep architectures including sleep stages, effect of positions on sleep, REM and non-REM AHI, and arousal index	No	Yes	Yes	Yes	Yes	No	Yes	Yes

Walsh et al., 2011 [17] USA	18-year-olds with OSA symptoms who refused or are nonadherent to CPAP with less than 3 hours of nightly use & AHI on baseline of >15 or >10 with symptoms of daytime sleepiness, mood issues, or hypertension or cognitive impairment	Nasal blockage or allergies or sinusitis, chronic use of nasal decongestants or nasal lesions, comorbid sleep, other sleep disorders, cardiorespiratory diseases, psychiatric disorders, pregnancy, and large caffeine consumption	AHI, ODI, ESS, FOSQ, and sleep architecture variables including sleep stages, percent of oxygen saturations, and arousal indices	No	Yes	Yes	Yes	Yes	No	Yes	Yes

Rossi et al., 2013 [14] UK, Switzerland	Diagnosed and treated for OSA with CPAP for >12 months of ≥ 4 hours of nightly use prior to this trial in ages between 20 and 75 years, ODI of >10/h on baseline PSG or >10/h on nocturnal pulse oximetry test prior to enrolling for this trial. This test was performed on last night of 4-day CPAP off period	H/o CAD, PAD, uncontrolled or severe HTN, ventilator failure or Cheyne-Stokes breathing, sleep related accidents, comorbid sleep disorder, or commercial drivers	AHI, ODI, ESS, BP, and ODI from home oximetry	Yes	Yes	Yes	Yes	Yes	Yes	Yes	Yes

Friedman et al., 2015 [15] USA	Age >18, diagnosis of OSA in previous 12 mo., failed CPAP	No stringent exclusion criteria	AHI, ODI, SPO_2_, snoring by VAS, ESS, and SAQLI	No	Yes	Yes	Yes	Yes	No	Yes	Yes

^**∗**^Quality assessment of the included studies checklist from questions from National Institute for Health and Clinical Excellence (NICE): 1–8: (1) Case series collected in more than one center? (2) Is the hypothesis/aim/objective of the study clearly described? (3) Are the inclusion and exclusion criteria clearly reported? (4) Is there a clear definition of the outcomes reported? (5) Were data collected prospectively? (6) Is there an explicit statement that patients were recruited consecutively? (7) Are the main findings of the study clearly described? (8) Are outcomes stratified?

PLMD: periodic limb movement disorder; MI: myocardial infarction or angina; PAP: positive airway pressure; B/W: between; ODI: oxygen desaturation index; min SPO_2_: minimum oxygen saturation; TST SPO_2_: total sleep time with oxygen saturation; PSQI: Pittsburgh Sleep Quality Index; ESS: Epworth Sleepiness Scale; COPD: chronic obstructive pulmonary disease; CHF: congestive heart failure; REM: rapid eye movement sleep; non-REM: nonrapid eye movement sleep; FEV1: forced expiratory volume in 1 second; FVC: forced vital capacity; FRC: functional residual capacity; ERV: expiratory reserve volume; TLC: total lung capacity; *P*_crit_: critical closing pressure; EtCO_2_: end tidal CO_2_; n-EPAP: nasal EPAP (Provent); FOSQ: Functional Outcomes of Sleep Questionnaire; CAD: coronary artery disease; PAD: peripheral arterial disease; HTN: hypertension; BP: blood pressure; SAQLI: sleep apnea quality of life index; VAS: visual analog scale for snoring measurement.

**Table 3:** Pre- and postnasal EPAP means, standard deviations, mean differences, and confidence intervals for polysomnography outcomes.

Research studies	Study type	Patient characteristics			Apnea-hypopnea index (mean/SD, except^∧^)				Epworth Sleepiness Score (mean/SD except^∧^)			
Author, year		*N*	Age, years	BMI Kg/m^2^	Pre-NEPAP	Post-NEPAP	MD (95% CI)	*p* value	Pre-NEPAP	Post-NEPAP	MD (95% CI)	*p* value
Colrain et al., 2008 [12]	Original	30	50.1 ± 9.8	28.2 ± 4.0	24.8 ± 22.1	14.2 ± 21.8	−10.60 (0.745, −21.945)	0.0665	—	—	—	—
			49 ± 12.9F	27 ± 5.34F								
Rosenthal et al., 2009 [16]	Original	28	49.8 ± 10.2	30.1 ± 5.9	24.5 ± 23.6	15.5 ± 18.9	−9.00 (2.456, −20.456)	0.1211	8.7 ± 4.0	6.9 ± 4.4	−1.80 (0.453, −4.053)	0.115
Hwang et al., 2009 [20]	C. abstract	6	26–39	—	8 to 97	<20 in 3 pts	—	—	—	—	—	—
Westbrook et al., 2009 [21]	C. abstract	58	—	—	26.6 ± 24.8	13.7 ± 20.1	−12.90 (−4.596, −21.204)	0.0026	—	—	—	—
Berry et al., 2011^∧^ [18]	Original	229	47.7 ± 13.4	32.6 ± 7.0	14.4	5.6	—	—	9.9 ± 4.7	7.2 ± 4.2	−2.70 (−1.881, −3.519)	0.0001
Kryger et al., 2011^∧^ [19]	Original	34	50.1 ± 13.6	32.5 ± 7.5	15.7	4.7	—	—	14	7	—	—
Patel et al., 2011 [13]	Original	19	54.3 ± 12.0	33.5 ± 5.6	34 ± 30.0	19.9 ± 26.0	−14.10 (4.371, −32.571)	0.1303	—	—	—	—
Walsh et al., 2011 [17]	Original	43	53.7 ± 10.9	34.9 ± 6.7	43.3 ± 29.0	16.4 ± 12.2	−26.90 (−17.359, −36.441)	0.0001	12.3 ± 4.8	8.7 ± 4.4	−3.60 (−1.625, −5.575)	0.0005
Adams 2011^∧^ [22]	C. abstract	131	—	—	25.8	4.2	—	—	—	—	—	—
Hwang et al., 2011 [23]	C. abstract	36	56.3 ± 12.6	34.1 ± 9.5	22.7 ± 19.4	8.9 ± 9.9	−13.80 (−6.560, −21.040)	0.0003	—	—	—	—
Massie and Hart 2011^∧^ [24]	C. abstract	30	51 ± 10	32 ± 6	17.1	4.9	—	—	7.2^*∗∗*^	5.5^*∗∗*^	—	—
Adams 2012^∧^ [25]	C. abstract	72	≥65	—	26.3	4.7	—	—	—	—	—	—
Massie 2012^∧^ [26]	C. abstract	31	—	—	17.5	5	—	—	10.6	5.9	—	—
Rossi et al., 2013 [14]	Original	22	63.2 ± 8.7	33.4 ± 6.6	36.5 ± 15.1	27.6 ± 16.4	−8.90 (0.692, −18.492)	0.0681	7 ± 5.8–9.0	9.3 ± 4.8	2.30 (5.620, −1.020)	0.1692
Deoras et al., 2013^*∗∗∗*^ [27]	C. abstract	42	≥18	—	—	−22.4 ± 21	—	—	9.8	7.6	—	—
Mansfield et al., 2013 [28]	C. abstract	21	—	—	32 ± 5.0	9.0 ± 6.0	−23.00 (−19.56, −26.44)	0.0001	7 ± 10	6 ± 11	−1.00 (5.56, −7.56)	0.7595
Martin et al., 2014 [29]	C. abstract	50	—	—	19.3 ± 12.6	5.5 ± 13.1	−13.80 (−8.699, −18.901)	0.0001	—	—	—	—
Friedman et al., 2015 [15]	Original	38	51.5 ± 11.6	29.4 ± 5.0	18.1 ± 13.0	7.4 ± 10.1	−10.70 (−5.379, −16.021)	0.0001	10.1 ± 5.5	8.5 ± 5.0	−1.60 (0.803, −4.003)	0.1886
Total patients		920										
Combined means^#^			50.2 ± 12.7	32.2 ± 6.7	*27.32 ± 22.24*	*12.78 ± 16.89*	−*14.54 (*−*17.59, *−*11.49)*	*<0.0001*	*9.94 ± 5.29*	*7.42 ± 4.98*	−*2.52 (*−*1.766, *−*3.273)*	*<0.0001*

^*∗∗*^21 patients.

^*∗∗∗*^Mean change in AHI.

^#^Combined means of studies included in meta-analysis.

^∧^Median (25–75).

NEPAP: nasal EPAP (Provent); SD: standard deviation; C. abstract: conference abstract; F: female; Kg/m^2^: kilogram per square meter.

**Table 4:** Pre- and postnasal EPAP means, standard deviations, mean differences, and confidence intervals for polysomnography outcomes.

Research studies	Oxygen desaturation index (mean/SD, except^∧^)				Lowest oxygen saturation (mean/SD except^∧^)			
Author, year	Pre-NEPAP	Post-NEPAP	MD (95% CI)	*p* value	Pre-NEPAP	Post-NEPAP	MD (95% CI)	*p* value
Colrain et al., 2008 [12]	14.6 ± 16.9	9.9 ± 15.2	−4.70 (−13.007, 3.607)	0.2621	82.6 ± 8.6	85.3 ± 8.4	2.70 (1.693, 7.093)	0.2236
Rosenthal et al., 2009 [16]	11.0 ± 17.5	9.2 ± 14.3	−1.80 (−10.363, 6.763)	0.6751	85.0 ± 7.4	85.1 ± 8.1	0.10 (−3.96, 4.16)	0.9617
Hwang et al., 2009 [20]	—	—	—	—	—	—	—	—
Westbrook et al., 2009 [21]	—	—	—	—	—	—	—	—
Berry et al., 2011^∧^ [18]	12.6	8.6	—	—	—	—	—	—
Kryger et al., 2011^∧^ [19]	12.6	7.6	—	—	—	—	—	—
Patel et al., 2011 [13]	—	—	—	—	—	—	—	—
Walsh et al., 2011 [17]	38.8 ± 27.2	17.0 ± 11.3	−21.80 (−30.732, −12.868)	0.0001	—	—	—	—
Adams 2011^∧^ [22]	—	—	—	—	—	—	—	—
Hwang et al., 2011 [23]	21.8 ± 13.0	12.1 ± 10.3	−9.70 (−15.213, −4.187)	0.0008	—	—	—	—
Massie and Hart 2011^∧^ [24]	18.8^*∗*^	4.4^*∗*^	—	—	—	—	—	—
Adams 2012^∧^ [25]	—	—	—	—	—	—	—	—
Massie 2012^∧^ [26]	13.7	8.2	—	—	—	—	—	—
Rossi et al., 2013 [14]	33.1 ± 12.2	35.8 ± 17.4	2.70 (−6.672, 12.072)	0.5637	—	—	—	—
Deoras et al., 2013 [27]	—	—	—	—	—	—	—	—
Mansfield et al., 2013 [28]	—	—	—	—	—	—	—	—
Martin et al., 2014 [29]	13.8 ± 11.4	5.0 ± 5.8	−8.80 (−12.4, −5.3)	<0.00001	84.0 ± 5.0	86.7 ± 16.2	2.70 (−2.0, 7.45)	0.2629
Friedman et al., 2015 [15]	16.3 ± 10.8	8.2 ± 9.0	−8.10 (−12.644, −3.556)	0.0007	81.3 ± 6.7	86.9 ± 5.6	5.60 (2.777, 8.423)	0.0002
Combined means^#^	21.2 ± 19.3	12.4 ± 14.1	−8.80 (−11.78, −5.82)	<0.00001	83.2 ± 6.8	86.2 ± 11.1	3.00 (0.89, 5.11)	0.005

^*∗*^16 patients.

^#^Combined means of studies included in meta-analysis.

^∧^Median (25–75).

NEPAP: nasal EPAP (Provent); SD: standard deviation.
